# Cross-sectional survey in CKD patients across Europe describing the association between quality of life and anaemia

**DOI:** 10.1186/s12882-016-0312-9

**Published:** 2016-07-26

**Authors:** Daniel Eriksson, David Goldsmith, Siguroli Teitsson, James Jackson, Floortje van Nooten

**Affiliations:** 1 Quantify Research, Stockholm, Sweden; 2 Renal Unit, Guy’s and St Thomas’ NHS Foundation Hospital, London, UK; 3 Adelphi Real World, Macclesfield, Cheshire, UK; 4 Formerly with Astellas Pharma, Leiden, The Netherlands

**Keywords:** Anaemia, Chronic kidney disease, EQ-5D, Fatigue, KDQOL-36, Quality of life, SF-12, Utility

## Abstract

**Background:**

Deteriorating renal function in chronic kidney disease (CKD) patients is commonly associated with reduced haemoglobin levels, adding to the already considerable humanistic burden of CKD. This analysis evaluated the impact of anaemia on disease burden in patients with CKD stages 3–4, and in those on dialysis.

**Methods:**

This was a descriptive, cross-sectional analysis of European data from an Adelphi CKD Disease-Specific Programme. This programme collected data from patients and their treating nephrologists/endocrinologists; patient- and physician-reported data were matched for each patient. Health-related quality of life (HRQoL) data were obtained through patient completion of the EQ-5D, SF-12 and KDQOL-36. Additional information was obtained via physician reporting of patient symptoms, and patients’ reports of impaired activity. Anaemia was defined by haemoglobin level and/or current use of erythropoiesis stimulating agents.

**Results:**

Significant, but modest Spearman’s rank correlations were observed between haemoglobin levels and extent of HRQoL impairment, regardless of instrument used (range 0.19–0.23; all *P*-values < 0.0001). When stratified by anaemia status, impairment was consistently lower for anaemic than non-anaemic CKD patients across measurement scales (e.g. EQ-5D index value [standard deviation {SD}] 0.72 [0.31] vs 0.83 [0.23], respectively; *P* < 0.0001). Physician-reported patient tiredness was associated with increased disease burden at all levels of CKD studied (total EQ-5D index value [SD] in patients reporting no tiredness vs tiredness 0.81 [0.26] vs 0.70 [0.30] respectively; *P* < 0.0001) with *P* < 0.0001 for no tiredness vs tiredness at all stages of CKD. The presence of anaemia was associated with impaired activity levels at CKD stages 3 (37.5 % vs 28.4 %, respectively; *P* = 0.0044) and 4 (48.1 % vs 39.9 %, respectively; *P* = 0.0292), and in patients on dialysis (52.0 % vs 45.0 %, respectively; *P* = 0.0732).

**Conclusions:**

The analysis found that CKD patients with anaemia typically had a lower HRQoL than those without anaemia. The impairment correlated with anaemia was more apparent in non-dialysis patients with CKD stages 3 or 4 than in those receiving dialysis. Coexisting CKD and anaemia may have an impact on patient HRQoL similar to other chronic conditions such as diabetes, epilepsy or certain forms of cancer.

**Electronic supplementary material:**

The online version of this article (doi:10.1186/s12882-016-0312-9) contains supplementary material, which is available to authorized users.

## Background

It is well established that patients with chronic kidney disease (CKD) are at increased risk of developing anaemia; both the prevalence and severity of anaemia correlate with the progressive decline in estimated glomerular filtration rate (eGFR) [1, 2]. A cross-sectional analysis of data from the National Health and Nutrition Examination Survey (NHANES) in the US reported that anaemia was twice as prevalent in people with CKD (15.4 %) as in the general population (7.6 %), and that the prevalence increased with the stage of CKD from 8.4 % at stage 1 to 53.4 % at stage 5 [3]. The NHANES study used the same definition of anaemia as recommended for adult patients with CKD by the Kidney Disease Improving Global Outcomes (KDIGO) Clinical Practice Guideline for Anaemia in Chronic Kidney Disease, namely serum haemoglobin < 12 g/dL in women and < 13 g/dL in men [2]. A range of comorbidities, such as cardiovascular disease, cognitive impairment, hospitalisation and mortality, are associated with anaemia in CKD [4, 5]. In some cases, the risk of anaemia in CKD may be elevated by the presence of such comorbidities; for example, it has been reported that anaemia is more common in diabetic patients with CKD stages 3 or 4 than in non-diabetic patients with similar renal function [6].

Anaemia contributes to the impairment of health-related quality of life (HRQoL) in patients with CKD [7]. Its impact on patients’ HRQoL burden is exacerbated by reduced physical capacity and energy levels among these patients. Anaemia is linked with a decline in physical function, independently of its association with renal impairment, which can reduce a patient’s ability to perform activities associated with daily living [8]. A recent systematic review explored the humanistic burden, reflected in impaired HRQoL and other patient-reported outcomes, associated with anaemia in patients with CKD [9]. This review confirmed that those with lower haemoglobin or haematocrit levels had a poorer HRQoL based on the 36-Item Short Form Health Survey (SF-36) compared with CKD patients with higher levels, and that this trend held whether or not patients were receiving dialysis. The findings also highlighted an inverse association between the severity of anaemia and scores on the physical and mental component summaries of the SF-36.

The aim of this survey was to evaluate the impact of anaemia on the burden of disease in patients with different stages of CKD, including those patients on dialysis. The findings from this analysis can provide an important comparison for the HRQoL of patients with CKD and anaemia relative to that of patients with other chronic diseases.

## Methods

### Study design

This was a descriptive cross-sectional analysis. The data on which the analyses were performed were derived from an existing Adelphi CKD Disease-Specific Programme (DSP), which was conducted by Adelphi Real World (Macclesfield, UK) between June and September 2012 in France, Germany, Italy, Spain and the UK. DSPs are large, multi-national, cross-sectional surveys of clinical practice, and incorporate patient and physician-reported data on specific chronic diseases. The full methodology for the conduct of DSPs has been published previously [10].

Participating physicians were nephrologists or endocrinologists who were actively involved in the management of CKD. Following recruitment after a clinical interview, physicians were asked to complete a detailed Patient Record Form (PRF) for consecutive patients they were managing for CKD. The physicians were asked to provide information for the next 8 non-dialysis patients with CKD, and 4–12 (depending on the country) dialysis patients with CKD. For endocrinologists, recruitment criteria required that 14 non-dialysis patients (or 12 patients in the UK) were seen consecutively. Physicians asked all patients for whom a PRF was completed whether they were willing to complete a patient self-completion form (PSC). Patients provided informed consent via a tick box on the front of the PSC questionnaire. The data were collected according to market research guidelines; hence, no source validation was possible or required. Patient and doctor identities were not known to Adelphi. There were no identifiers recorded for the patients themselves. Patient and physician forms for the same ‘matched’ patients were linked by unique numeric codes preprinted on the questionnaires.

### Data collection

Physician-completed PRFs requested general information about patients and their CKD history, treatments, comorbidities, symptoms and performance status, along with healthcare resource utilisation. Patients independently completed PSC questionnaires that requested complementary information on their CKD history, healthcare resource utilisation, work productivity and HRQoL. The PSC and PRF data were linked for each patient.

### Outcome measures

While the PSC and PRF forms enabled collection of data on a range of outcomes, this analysis was designed particularly to focus on data relating to patients’ HRQoL. In the PSC questionnaire, patients’ HRQoL was primarily assessed using the EuroQol Group’s generic EQ-5D-3 L utility score, with additional information obtained via the disease-specific Kidney Disease and Quality of Life Instrument (KDQOL-36). The EQ-5D-3 L is a descriptive system of patient HRQoL states consisting of five dimensions (mobility, self-care, usual activities, pain/discomfort and anxiety/depression), each of which can take one of three responses (no problems, some or moderate problems, and extreme problems) [11]. KDQOL-36 comprises subscales for the burden of kidney disease, symptoms and problems, and the effects of kidney disease on daily life, including the 12-Item Short Form Health Survey (SF-12) measures of physical and mental functioning [12, 13]. SF-12 is a shorter version of the more established SF-36 tool for measuring health outcomes [14].

The symptoms of anaemia as documented by physicians on the PRF were also relevant to the analyses; physicians were asked to report symptoms of tiredness/low energy, lethargy and fatigue in their patients. In addition, information on the perceptions of general activity impairment was obtained via the Work Productivity and Activity Impairment (WPAI) questionnaire, which was incorporated in the PSC [15].

### Statistical analyses

Analyses were performed on the overall patient population and by disease severity. Patients were stratified into three levels of disease severity: CKD stages 3 (30 ≤ eGFR < 60 mL/min/1.73 m^2^), 4 (15 ≤ eGFR < 30 mL/min/1.73 m^2^), and dialysis. Anaemia was defined based on low haemoglobin levels (KDIGO definition: serum haemoglobin < 12 g/dL in women and < 13 g/dL in men) and/or current use of erythropoiesis stimulating agents (ESAs).

The relationship between haemoglobin level and HRQoL was evaluated using the non-parametric Spearman’s rank correlation. The Wilcoxon-Mann-Whitney test, Kruskal-Wallis test and *χ*^2^-test were used as applicable. All analyses were conducted in Stata/IC version 12.1 for Windows (StataCorp LP, College Station, TX, US).

## Results

### Patient characteristics

A total of 242 physicians (216 nephrologists and 26 endocrinologists) participated in the Adelphi CKD DSP survey (France: 50, Germany: 53, Italy: 50, Spain: 52, United Kingdom: 37). Recruitment of patients into the DSP by these physicians generated a pool of 2986 patients with an available eGFR or dialysis status (from 3057 patients enrolled in total). Of these, 1873 were stage 3 or 4 non-dialysis CKD patients (1039 stage 3; 834 stage 4) and 1025 were receiving dialysis, thus providing a combined evaluable population for the survey of 2898 patients. A total of 1336 completed PSC questionnaires were received. Patients who did not complete a questionnaire tended to be slightly older (64.5 compared with 62.8 years old), had been diagnosed with CKD an average of 7 months longer, and were slightly less likely to be on dialysis (32 % vs. 39 %). There were only minor differences in the underlying cause of condition between questionnaire completers and non-completers.

The patient characteristics are summarised in Table 1. The four most common causes of CKD reported in stage 3, stage 4 and dialysis patients were hypertension (53, 60 and 44 %, respectively), type 2 diabetes (43, 42 and 32 %, respectively), cardiovascular morbidities (18, 21 and 15 %, respectively) and glomerulonephritis (13, 14 and 19 %, respectively). Mean (± standard deviation [SD]) haemoglobin levels were lower in patients on dialysis than in those with CKD stages 3 or 4 (11.2 ± 1.5 vs 11.5 ± 1.5 vs 12.5 ± 1.7 g/dL, respectively; *P* = 0.0001 for a difference between the groups), which is consistent with the established links between CKD severity and risk and severity of anaemia. Among the non-dialysis patients, eGFR values were also lower in anaemic than non-anaemic patients (29.6 ± 10.7 vs 37.5 ± 11.3 mL/min/1.73 m^2^; *P* < 0.0001). Of the patients on dialysis, 78 % were receiving an ESA and/or supplemental iron, compared with 50 % of those with CKD stage 4 and only 19 % of those with CKD stage 3.

**Table 1:** Patient characteristics

	CKD stage 3 (*n* = 1039)	CKD stage 4 (*n* = 834)	Dialysis (*n* = 1025)	Total (*n* = 2898)
Sex, *n* (%)				
Female	411 (40)	343 (41)	410 (40)	1164 (40)
Male	628 (60)	491 (59)	615 (60)	1734 (60)
Age, years				
Mean ± SD	63.4 ± 14.7	66.9 ± 14.5	61.5 ± 15.4	63.7 ± 15.1
≥65, *n* (%)	548 (53)	535 (64)	490 (48)	1573 (54)
Body mass index, kg/m^2^				
Mean ± SD	26.8 ± 4.7	27.0 ± 5.3	25.7 ± 4.9	26.5 ± 5.0
≥30, *n* (%)	210 (20)	178 (21)	155 (15)	543 (19)
˂ 30, *n* (%)	792 (76)	624 (75)	822 (80)	2238 (77)
Missing	37 (4)	32 (4)	48 (5)	117 (4)
Anaemia status, *n* (%)				
Anaemic	453 (44)	633 (76)	887 (87)	1973 (68)
Non-anaemic	479 (46)	177 (21)	84 (8)	740 (26)
Missing	107 (10)	24 (3)	54 (5)	185 (6)
ESA and/or iron use, *n* (%)	197 (19)	420 (50)	801 (78)	1418 (49)
Country, *n* (%)				
France	206 (20)	177 (21)	190 (19)	573 (20)
Germany	238 (23)	168 (20)	306 (30)	712 (25)
Italy	214 (21)	161 (19)	190 (19)	565 (20)
Spain	232 (22)	182 (22)	190 (19)	604 (21)
UK	149 (14)	146 (18)	149 (15)	149 (15)
Comorbidities, *n* (%)				
Hypertension	903 (87)	741 (89)	725 (71)	2369 (82)
SHPT	256 (25)	476 (57)	688 (67)	1420 (49)
Type 2 diabetes	457 (44)	355 (43)	335 (33)	1147 (40)
Dyslipidaemia	448 (43)	365 (44)	335 (33)	1148 (40)
Employment status, *n* (%)				
Employed ^a^	288 (28)	142 (17)	199 (19)	629 (22)
Retired	535 (51)	511 (61)	539 (53)	1585 (55)
Other ^b^	216 (21)	181 (22)	287 (28)	684 (24)

*Abbreviations*: *CKD* chronic kidney disease, *ESA* erythropoiesis stimulating agent, *SD* standard deviation, *SHPT* secondary hyperparathyroidism

^a^ Full-time, part-time, or self-employed

^b^ Unemployed, homemaker, student, other

Some country-specific differences were noted in baseline characteristics. The proportion of patients on dialysis varied from 31 % in Spain to 43 % in Germany, and mean age ranged from 61 years (UK) to 66 years (France). The proportion of patients that were 65 or older ranged from 49 % for Germany and the UK to 64 % for France. Mean EQ-5D ranged from 0.63 (France) to 0.85 (Italy).

### Quality of life—overview

**Fig. 1:**
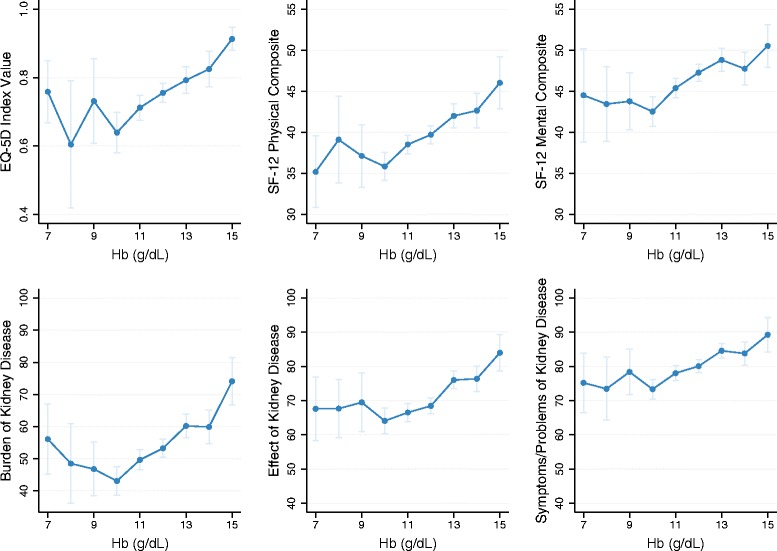
HRQoL measures by serum haemoglobin level. Significant but modest Spearman’s correlation coefficients between HRQoL measures and Hb (range 0.19–0.23; all *P*-values < 0.0001). Hb levels recorded on the x-axis represent the midpoint of the Hb range (e.g. 7 g/dL refers to levels 6.5 ≤ Hb < 7.5 g/dL). Vertical lines represent the 95 % confidence interval around the mean. EQ-5D, *n* = 1147; SF-12, *n* = 1086; burden of kidney disease, *n* = 1169; effect of kidney disease, *n* = 1149; symptoms of/problems with kidney disease, *n* = 1140. Hb, haemoglobin; SF-12, 12-Item Short Form Health Survey; HRQoL, health-related quality of life

Significant correlations were observed between serum haemoglobin levels and disease burden; the relationship was similar regardless of whether the burden was measured using EQ-5D index scores, SF-12 physical and mental composite summary scores, or the three other subscales of the KDQOL-36 scale (Fig. 1).

### EQ-5D

The overall EQ-5D index value across the patient groups was 0.76, and EQ-5D values were consistently lower for anaemic vs non-anaemic CKD patients (Table 2).

**Table 2:** EQ-5D index value by anaemia status by stages of CKD

Disease severity	Non-anaemic (*n* = 313) Mean (SD)	Anaemic (*n* = 864) Mean (SD)	*P*-value
CKD stage 3	0.85 (0.21)	0.78 (0.29)	0.0071
CKD stage 4	0.81 (0.22)	0.71 (0.28)	0.0006
Dialysis	0.74 (0.29)	0.70 (0.32)	0.4082
Total	0.83 (0.23)	0.72 (0.31)	<0.0001

*Abbreviations*: *CKD* chronic kidney disease, *SD* standard deviation

**Fig. 2:**
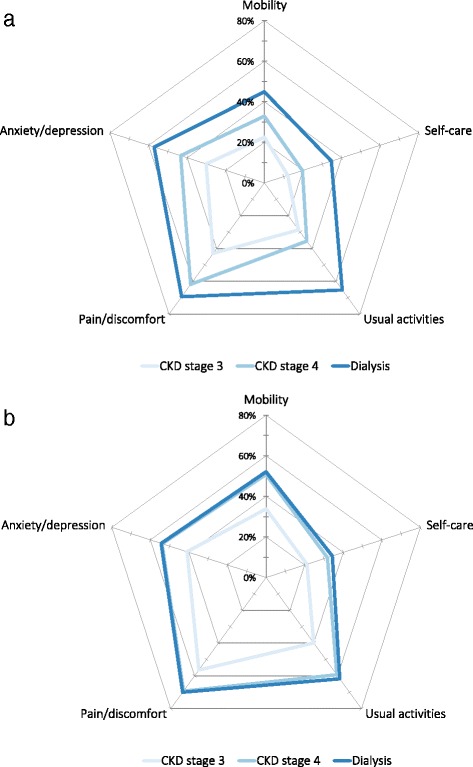
Proportion of patients reporting problems for the five EQ-5D dimensions by stages of chronic kidney disease. **a** Non-anaemic patients and **b** anaemic patients. CKD, chronic kidney disease

Problems were more pronounced for patients on dialysis than those with less severe CKD (Table 3; see also Additional file 1: Table S1 and S2 for breakdown by CKD stage), when analysed by the five separate dimensions of EQ-5D. However, the difference in the extent of these problems between patients on dialysis and those with CKD stage 4 was considerably smaller in anaemic than in non-anaemic patients (Fig. 2).

**Table 3:** Proportion of patients reporting problems for the five EQ-5D dimensions by dialysis status

EQ-5D dimension	CKD stages 3–4 *n* (%)	Dialysis *n* (%)	*P*-value
Mobility	278 (35)	247 (48)	<0.0001
Self-care	169 (21)	166 (33)	<0.0001
Usual activities	325 (41)	304 (60)	<0.0001
Pain/discomfort	437 (56)	346 (68)	<0.0001
Anxiety/depression	323 (41)	272 (53)	<0.0001

*Abbreviations*: *CKD* chronic kidney disease

### Other measures of quality of life

Scores on the SF-12 physical and mental composite scales, and the three additional subscales of the KDQOL-36, demonstrated similar trends in EQ-5D scores. Impairment was significantly greater among patients with anaemia than those without (Table 4). The only patients for whom mean scores were not lower in the anaemic group were those on dialysis (based on SF-12 mental composite summary scores).

**Table 4:** Health-related quality of life scores by anaemia status in patients with CKD stages 3 and 4 and those on dialysis

Subscales of the KDQOL-36	Non-anaemic Mean (SD)	Anaemic Mean (SD)	*P*-value
Symptoms/problems list	84.9 (15.6)	78.2 (18.0)	<0.0001
Effect of Kidney Disease	78.7 (17.7)	67.0 (21.3)	<0.0001
Burden of Kidney Disease	63.6 (25.7)	49.9 (26.3)	<0.0001
SF-12 physical composite summary	43.1 (9.8)	38.5 (9.9)	<0.0001
CKD stage 3	45.1 (9.1)	42.3 (9.9)	0.0112
CKD stage 4	40.6 (10.2)	38.0 (9.5)	0.0506
Dialysis ^a^	39.5 (10.3)	37.3 (9.8)	0.1319
SF-12 mental composite summary	48.5 (9.1)	45.8 (10.0)	<0.0001
CKD stage 3	50.6 (8.2)	47.5 (9.5)	0.0028
CKD stage 4	46.7 (9.3)	45.5 (9.8)	0.2841
Dialysis ^a^	43.9 (9.1)	45.2 (10.3)	0.3507

*Abbreviations*: *CKD* chronic kidney disease, *KDQOL*-*36* Kidney Disease Quality of Life Instrument, *SD* standard deviation, *SF*-*12*, 12-Item Short Form Health Survey

^a^ Note: Anaemia in dialysis patients may be affected by level of erythropoiesis stimulating agent/iron use (78 % of dialysis patients vs 50 % in CKD stage 4 and 19 % in CKD stage 3, respectively)

Further analysis of the SF-12 physical and mental composite summary scores, and the three additional subscales of the KDQOL-36, revealed that the use of ESAs and/or iron supplementation was associated with lower mean scores (Table 5).

**Table 5:** Health-related quality of life scores by erythropoiesis stimulating agent/supplemental iron use in patients with CKD stages 3 and 4 and those on dialysis

Subscales of the KDQOL-36	No ESA/supplemental iron use Mean (SD)	ESA and/or supplemental iron use Mean (SD)	*P*-value
Symptoms/problems list	83.5 (17.1)	77.1 (17.6)	<0.0001
Effect of Kidney Disease	76.9 (19.0)	64.5 (21.0)	<0.0001
Burden of Kidney Disease	61.5 (25.8)	46.9 (25.8)	<0.0001
SF-12 physical composite summary	42.4 (10.1)	37.6 (9.5)	<0.0001
CKD stage 3	44.6 (9.3)	40.8 (9.9)	0.0019
CKD stage 4	40.5 (10.3)	36.9 (8.9)	0.0007
Dialysis	39.0 (10.4)	37.1 (9.7)	0.1441
SF-12 mental composite summary	47.9 (9.3)	45.4 (10.1)	<0.0001
CKD stage 3	49.7 (8.6)	46.9 (10.0)	0.0206
CKD stage 4	46.9 (9.4)	44.9 (9.8)	0.0653
Dialysis	44.3 (10.0)	45.3 (10.3)	0.4347

*Abbreviations*: *CKD* chronic kidney disease, *ESA* erythropoiesis stimulating agent, *KDQOL-36* Kidney Disease Quality of Life Instrument, *SD* standard deviation, *SF-12* 12-Item Short Form Health Survey

### Tiredness in patients with CKD and anaemia

Tiredness and low energy were reported in 31 % of patients with CKD stage 3, 50 % of patients with CKD stage 4 and 47 % of dialysis patients. Reports of lethargy (8, 17 and 14 %, respectively) or fatigue (24, 39 and 34 %, respectively) were less common. As expected, tiredness was more common among anaemic than non-anaemic patients (49 % vs 30 %, respectively; *P* < 0.0001).

**Fig. 3:**
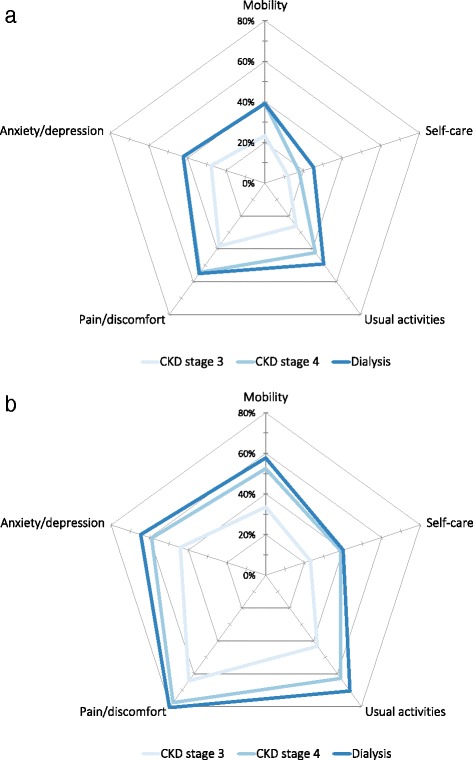
Proportion of patients reporting problems for the five EQ-5D dimensions by stages of chronic kidney disease. **a** Patients without tiredness symptoms (*n* = 710) and **b** patients with tiredness symptoms (*n* = 577). CKD, chronic kidney disease

Owing to the importance of tiredness as a symptom for patients with anaemia, further analysis of EQ-5D data was performed with stratification by patient tiredness. Patients with tiredness had more problems across all five EQ-5D dimensions than those without tiredness, regardless of CKD severity (Fig. 3). Similarly, the overall EQ-5D index value was lower in patients with tiredness than without, whether patients were CKD stage 3 (0.78 vs 0.85, respectively; *P* < 0.0001), CKD stage 4 (0.68 vs 0.80, respectively; *P* < 0.0001) or on dialysis (0.66 vs 0.77, respectively; *P* < 0.0001).

### Patient assessments of activity impairment

Activity impairment, as assessed by the WPAI, was greater in anaemic (*n* = 751) than in non-anaemic (*n* = 277) patients, at CKD stage 3 (37.5 % vs 28.4 %, respectively; *P* = 0.0044), CKD stage 4 (48.1 % vs 39.9 %, respectively; *P* = 0.0292) or on dialysis (52.0 % vs 45.0 %, respectively; *P* = 0.0732). The extent of activity impairment correlated inversely with serum haemoglobin levels (ρ = -0.28; *P* < 0.0001).

## Discussion

This descriptive analysis of cross-sectional data obtained through the Adelphi DSP for CKD evaluated the characteristics of enrolled patients and confirmed the recognised link between anaemia and CKD, with an inverse association observed between serum haemoglobin levels and CKD severity, as well as lower eGFR levels in anaemic vs non-anaemic patients.

The survey used several tools to assess the impact on patient utility and HRQoL. Significant correlation was observed between serum haemoglobin levels and disease burden, regardless of the assessment tool used. Accordingly, when analysed by anaemia status, impairment was generally greater in anaemic than non-anaemic patients with CKD across the measurement scales.

In this analysis, a noteworthy observation was that the effect of anaemia on patient HRQoL was smaller in dialysis than non-dialysis patients. In particular, the extent of problems across the five dimensions of the EQ-5D (Fig. 2), such as pain/discomfort, or difficulty in performing daily activities, suggests that the presence of anaemia in patients with CKD stage 4 accentuates the disease burden to levels more typically associated with patients on dialysis. Tiredness was found to increase the disease burden at all stages of CKD studied, with greater tiredness observed at later stages of CKD. Data also indicated that the presence of anaemia was associated with impaired activity levels across the stages of CKD studied.

When considering each of these aspects of the impact of anaemia on patients with CKD, it is worth emphasising the importance of taking a holistic view of these patients. Symptoms do not occur independently from one another. For example, examination of the data for individual dimensions of the EQ-5D indicates that problems with depression and anxiety are considerable among this patient population. Feelings of low mood and an impaired ability to perform daily activities may create a ‘downward spiral’ effect, with each symptom promoting the other. Partly as a result of this effect, it can be difficult to quantify the cause of some symptoms, or predict how these might improve if anaemia is treated. Decreased kidney function in CKD can be associated with complications such as cardiovascular disease, bone disease and cognitive impairment [16], although the link between CKD and subsequent cognitive impairment remains a complex subject of ongoing research [17]. Furthermore, anaemia has been reported to increase the risk of complications such as cognitive impairment and cardiovascular disease, independently of CKD [18, 19]. The tiredness associated with anaemia may limit a patient’s feelings of being able to cope with this multifaceted condition.

With regard to the question of anaemia correction, the findings of this survey show consistency with data from large studies such as the Cardiovascular Risk Reduction by Early Anemia Treatment with Epoetin Beta (CREATE) trial and the Correction of Hemoglobin and Outcomes in Renal Insufficiency (CHOIR) trial, which showed that correction of anaemia in non-dialysis patients with CKD was associated with improvements in patient HRQoL [20, 21]. However, it is also important to consider findings from the prospective Trial to Reduce Cardiovascular Events with Aranesp Therapy (TREAT) (patients with type 2 diabetes and stage 3/4 CKD) where darbepoetin alfa treatment generated only a modest improvement in patient-reported fatigue [22]. Importantly, the latter was a placebo-controlled trial, and is considered to have very important implications for the management of anaemia in CKD patients [23].

The quantitative data derived from this survey may be used to put the disease burden experienced by patients with CKD and anaemia into context with equivalent data from other chronic conditions. As a generic utility score, the EQ-5D index has been applied to numerous other chronic conditions, enabling some level of inter-study comparison. In this analysis, the mean EQ-5D index value for patients with CKD stage 3 and 4, or on dialysis, was 0.83 for those without anaemia, and 0.72 for those with anaemia. On this basis, the latter group have a similar level of impairment to patients with diabetes (0.73), as reported by a UK cohort study of 4485 patients in primary care [24]. Various other baseline EQ-5D values were reported by Peters et al. 2014, such as asthma (0.83), chronic obstructive pulmonary disease (0.67), epilepsy (0.76), heart failure (0.64) and stroke (0.67) [24]. Broadly similar EQ-5D values to those identified for CKD patients with anaemia have been reported, for example, with chronic hepatitis C (0.76; [25]), second-line metastatic colorectal cancer (pre- or post-progression 0.741 and 0.731, respectively; [26]) and rheumatoid arthritis (0.66; [27]). It is important to note that in CKD, the population means of such scores can be significantly influenced by the distribution of enrolled patients. For example, a higher proportion of dialysis patients would result in a correspondingly lower mean HRQoL score.

A particular strength of the survey was the demonstration of consistent findings across a number of patient HRQoL measures for the same patient sample. However, a number of limitations should also be acknowledged.

Clinical variables were not measured directly, which could lead to potential confounding. The definition of anaemia, which included low haemoglobin and/or use of ESAs, could have been a source of indication bias, as patients receiving ESAs were defined as having anaemia, irrespective of their haemoglobin levels. When the effect of anaemia on HRQoL was examined by ESA and/or iron use, it appeared that HRQoL scores were generally higher in those not receiving ESAs or iron supplementation.

Inter-country treatment practices were not reviewed, and therefore could not be controlled for. Due to the nature of patient selection (those attending consultation), caution should be used when extrapolating to the wider patient population. Although the overall enrolled population was substantial, some analyses relied on considerably smaller subgroups of patients; sample sizes are indicated in figure legends. Fewer than half of the patients with an available physician-reported eGFR or dialysis status voluntarily provided self-reported data via PSC questionnaires, which could have led to selection bias, as patients who did not complete a questionnaire tended to be slightly older, to have had a diagnosis CKD for a little longer, and were slightly more likely to be on dialysis. It is also worth considering that the questionnaires did not allow for explicit differentiation between instances of missing data and implied negative responses. For example, a question relating to whether a patient suffered from tiredness could only be answered via a confirmatory checkbox since no ’don’t know’ option was provided. Finally, no data were collected for a number of other factors that contribute to poor quality of life in patients with CKD, such as high inflammation, hypertension and vascular calcification; therefore adjustment for these potential confounding factors was not possible.

## Conclusions

Evidence suggests that correction of anaemia in patients with non-dialysis CKD significantly improves their HRQoL. In support of these reports, this analysis of the descriptive, cross-sectional survey described has demonstrated impaired HRQoL in patients with anaemia and CKD using a number of tools. QoL and haemoglobin were both lower among patients with dialysis, with the differences in QoL (observed between the instruments) potentially due to the dialysis treatment itself. The impairment that may be attributable to anaemia was greater in non-dialysis patients with CKD stages 3 or 4 than in those receiving dialysis; the presence of anaemia in patients with CKD stage 4 accentuated the disease burden to levels more typically associated with patients on dialysis. This indicates that correcting anaemia is particularly important for reducing burden in non-dialysis patients. In patients receiving dialysis, anaemia may already be well controlled, but being on dialysis itself contributes to overall disease burden, requiring a different treatment focus compared to non-dialysis patients. A close association between patients’ levels of tiredness and the impairment to their HRQoL indicated that this symptom may be a particularly important manifestation of anaemia with respect to the disease burden it imposes on patients. While no direct comparison was possible, consideration of data from studies of other chronic conditions indicates that patients with CKD and anaemia may experience a similar impact on their HRQoL to patients with diabetes, epilepsy or certain forms of cancer, such as metastatic colorectal cancer.

## Abbreviations

CKD, chronic kidney disease; CREATE, Cardiovascular Risk Reduction by Early Anemia Treatment with Epoetin Beta; DSP, Disease Specific Programme; eGFR, estimated glomerular filtration rate; ESA, erythropoiesis stimulating agent; HRQoL, health-related quality of life; KDIGO, Kidney Disease Improving Global Outcomes; KDQOL-36, Kidney Disease and Quality of Life Instrument; NHANES, National Health and Nutrition Examination Survey; PRF, patient record forms; PSC, patient self-completion; SD, standard deviation; SF-12/36, 12-/36-Item Short Form Health Survey; WPAI, Work Productivity and Activity Impairment

## Additional file

Additional file 1: Table S1. EQ-5D index value for patients with and without tiredness symptoms, by stages of CKD. Table S2. Proportion of patients reporting problems for the five EQ-5D dimensions by dialysis status and stage of CKD. (DOCX 13 kb)

## References

[1] Cheng CK, Chan J, Cembrowski GS, van Assendelft OW (2004). Complete blood count reference interval diagrams derived from NHANES III: stratification by age, sex, and race. Lab Hematol.

[2] KDIGO. Clinical Practice Guideline for the Evaluation and Management of Chronic Kidney Disease. Kidney Int 2013;3(1):1–150. Available from URL: http://www.kdigo.org/clinical_practice_guidelines/pdf/CKD/KDIGO_2012_CKD_GL.pdf. Accessed 20 Jul 2016. 10.1038/ki.2013.243 23989362

[3] Stauffer ME, Fan T (2014). Prevalence of anemia in chronic kidney disease in the united states. PLoS One.

[4] Li S, Foley RN, Collins AJ (2005). Anemia and cardiovascular disease, hospitalization, end stage renal disease, and death in older patients with chronic kidney disease. Int Urol Nephrol.

[5] Singh NP, Sahni V, Wadhwa A, Garg S, Bajaj SK, Kohli R (2006). Effect of improvement in anemia on electroneurophysiological markers (P300) of cognitive dysfunction in chronic kidney disease. Hemodial Int.

[6] Dousdampanis P, Trigka K, Fourtounas C (2014). Prevalence of anemia in patients with type II diabetes and mild to moderate chronic kidney disease and the impact of anti-RAS medications. Saudi J Kidney Dis Transpl.

[7] Okpechi IG, Nthite T, Swanepoel CR (2013). Health-related quality of life in patients on hemodialysis and peritoneal dialysis. Saudi J Kidney Dis Transpl.

[8] Bailey RA, Reardon G, Wasserman MR, McKenzie RS, Hord RS (2012). Association of anemia with worsened activities of daily living and health-related quality of life scores derived from the minimum data Set in long-term care residents. Health Qual Life Outcomes.

[9] Rizzo M, Iheanacho I, Van Nooten F, Goldsmith D (2014). A systematic literature review of the humanistic burden of anaemia associated with chronic kidney disease.

[10] Anderson P, Benford M, Harris N, Karavali M, Piercy J (2008). Real-world physician and patient behaviour across countries: disease-specific programmes - a means to understand. Curr Med Res Opin.

[11] EuroQol 2015 [accessed 2015 Feb 1]; Available from: URL: http://www.euroqol.org/

[12] Hays RD, Kallich JD, Mapes DL, Coons SJ, Carter WB (1994). Development of the kidney disease quality of life (KDQOL) instrument. Qual Life Res.

[13] Kidney disease quality of life-short form (KDQOL-SF) 2015 [accessed 2015 Feb 1]; Available from: URL: http://www.kdqol-complete.org/about/kdqol

[14] Short form-12 item questionaire (SF-12 2015) [accessed 2015 Feb 1]; Available from: URL: http://www.sf-36.org/tools/sf12.shtml.

[15] Reilly MC, Zbrozek AS, Dukes EM (1993). The validity and reproducibility of a work productivity and activity impairment instrument. Pharmacoeconomics.

[16] Jha V, Wang AY, Wang H (2012). The impact of CKD identification in large countries: the burden of illness. Nephrol Dial Transplant.

[17] McIntyre CW, Goldsmith DJ (2015). Ischemic brain injury in hemodialysis patients: which is more dangerous, hypertension or intradialytic hypotension?. Kidney Int.

[18] Pulignano G, Del SD, Di LA, Tinti MD, Tarantini L, Cioffi G (2014). Chronic renal dysfunction and anaemia are associated with cognitive impairment in older patients with heart failure. J Cardiovasc Med (Hagerstown).

[19] Zoppini G, Targher G, Chonchol M, Negri C, Stoico V, Pichiri I (2010). Anaemia, independent of chronic kidney disease, predicts all-cause and cardiovascular mortality in type 2 diabetic patients. Atherosclerosis.

[20] Drueke TB, Locatelli F, Clyne N, Eckardt KU, MacDougall IC, Tsakiris D (2006). Normalization of hemoglobin level in patients with chronic kidney disease and anemia. N Engl J Med.

[21] Singh AK, Szczech L, Tang KL, Barnhart H, Sapp S, Wolfson M (2006). Correction of anemia with epoetin Alfa in chronic kidney disease. N Engl J Med.

[22] Pfeffer MA, Burdmann EA, Chen CY, Cooper ME, Eckardt KU, De ZD (2009). A trial of darbepoetin Alfa in type 2 diabetes and chronic kidney disease. N Engl J Med.

[23] Goldsmith D, Covic A (2010). Time to reconsider evidence for anaemia treatment (TREAT) = essential safety arguments (ESA). Nephrol Dial Transplant.

[24] Peters M, Crocker H, Dummett S, Jenkinson C, Doll H, Fitzpatrick R (2014). Change in health status in long-term conditions over a one year period: a cohort survey using patient-reported outcome measures. Health Qual Life Outcomes.

[25] Samp JC, Perry R, Piercy J, Wood R, Baran RW (2015). Patient health utility, work productivity, and lifestyle impairment in chronic hepatitis C patients in France. Clin Res Hepatol Gastroenterol.

[26] Stein D, Joulain F, Naoshy S, Iqbal U, Muszbek N, Payne KA (2014). Assessing health-state utility values in patients with metastatic colorectal cancer: a utility study in the united kingdom and the Netherlands. Int J Colorectal Dis.

[27] Hernandez AM, Wailoo A, Wolfe F, Michaud K (2013). The relationship between EQ-5D, HAQ and pain in patients with rheumatoid arthritis. Rheumatology (Oxford).

[28] European Socioety for Opinion and Marketing Research (ESOMAR), International Code of Marketing and Social Research Practice 2007. Available from http://ethics.iit.edu/ecodes/node/5178. Accessed 20 Jul 2016.

