# Supplementary material for: Cross-sectional survey in CKD patients across Europe describing the association between quality of life and anaemia
Source: BMC Nephrol. 2016 Jul 26;17:97. doi: 10.1186/s12882-016-0312-9 (PMC4962379; doi:10.1186/s12882-016-0312-9)
Supplement: Additional file 1: Table S1. — EQ-5D index value for patients with and without tiredness symptoms, by stages of CKD. Table S2. Proportion of patients reporting problems for the five EQ-5D dimensions by dialysis status and stage of CKD. (DOCX 13 kb) [file 12882_2016_312_MOESM1_ESM.docx]

**Supplementary Table 1. EQ-5D index value for patients with and without tiredness symptoms, by stages of CKD**

| **Disease severity** | **No tiredness symptoms (n = 710)**  Mean (SD) | **Tiredness symptoms (n = 577)**  Mean (SD) | ***P*-value** |
| --- | --- | --- | --- |
| CKD stage 3 | 0.85 (0.24) | 0.78 (0.26) | < 0.0001 |
| CKD stage 4 | 0.80 (0.25) | 0.68 (0.29) | < 0.0001 |
| Dialysis | 0.77 (0.29) | 0.66 (0.33) | < 0.0001 |
| Total | 0.81 (0.26) | 0.70 (0.30) | < 0.0001 |
| CKD, chronic kidney disease; SD, standard deviation. | | | |

**Supplementary Table 2. Proportion of patients reporting problems for the five EQ-5D dimensions by dialysis status and stage of CKD**

| **EQ-5D dimension** | **CKD stage 3**  n (%) | **CKD stage 4**  n (%) | **Dialysis**  n (%) |
| --- | --- | --- | --- |
| Mobility | 118 (27) | 160 (46) | 247 (48) |
| Self-care | 69 (16) | 100 (29) | 166 (33) |
| Usual activities | 141 (32) | 184 (53) | 304 (60) |
| Pain/discomfort | 207 (45) | 230 (67) | 346 (68) |
| Anxiety depression | 147 (33) | 176 (51) | 272 (53) |
| CKD, chronic kidney disease. | | | |
